# Schwerpunkt: Das Superwahljahr 2021

**DOI:** 10.1007/s41358-021-00264-5

**Published:** 2021-05-27

**Authors:** Arno von Schuckmann

**Affiliations:** NRW School of Governance, Institut für Politikwissenschaft, Universität Duisburg-Essen, Lotharstraße 53, 47057 Duisburg, Deutschland

Superwahljahre sind selten. Sie zeichnen sich dadurch aus, dass besonders viele Wahlen in diesem einen Jahr stattfinden (Kommunalwahlen, Landtagswahlen und die Bundestagswahl). Superwahljahre sind kräftezehrend, denn die Parteien, deren Spitzenkandidaten, aber auch die Medien und die Gesellschaft befinden sich gefühlt in einem Dauerwahlkampf. Schließlich sind Superwahljahre prägend, denn die ersten Wahlen können bereits Vorboten für die nachfolgenden sein. Somit kann die politische Gemengelage nach einem solchen Jahr eine gänzlich andere sein als zuvor.

Als sei ein solches Wahljahr nicht schon Ereignis genug, trifft es zusammen mit der für die Bundesrepublik Deutschland, Europa und die Welt sicherlich größten Krise seit dem 2. Weltkrieg. Der Ausbruch der Covid-19-Pandemie führte der globalisierten Welt vor Augen, wie anfällig Gesundheit, Wirtschaft und auch Politik in einer immer weiter zusammenwachsenden Welt sind. Zunächst schien es in Deutschland so, als könne die pandemische Lage durch die beschlossenen Maßnahmen – wie den Lockdown – gut kontrolliert werden. Der vermeintliche deutsche Erfolg spiegelte sich auch in den herausragenden Umfragewerten der CDU/CSU wider und auch die Kanzlerin erfreute sich besonderer Beliebtheit. Zugleich zeigte sich in der Gesellschaft eine neue Faszination für die Wissenschaft. Hier standen insbesondere die Virologen und Epidemiologen nicht nur an der Seite der Politiker, sondern sie interpretierten auch die wenigen Daten für die Bevölkerung und gaben darauf basierend Verhaltensempfehlungen heraus. So prägten sie auch deutlich das Handeln der politischen Akteure. In großer Einheit zwischen Wissenschaft, Politik, Medien und Gesellschaft wurde unter dem Schlagwort *flatten the curve *versucht, die erste Welle der Pandemie einzudämmen.

Ein Jahr nach der ersten Welle befindet sich Deutschland im Frühjahr 2021 mitten in der dritten Welle der Corona-Pandemie. Jedoch gibt es einen entscheidenden Unterschied zur ersten Welle: Es gibt keine Einheit mehr. Die Bekämpfung des Virus folgt keiner einheitlichen Linie. Zwar wird immer wieder in langen Bund-Länder-Runden beraten, wie das Virus besiegt werden kann, dennoch werden die beschlossenen Maßnahmen wie Terminshopping, Schließung kultureller Einrichtungen, Home-Schooling oder gar eine nächtliche Ausgangsperre mit einer gewissen Regelmäßigkeit in den Ländern wieder aufgehoben oder nur in Teilen umgesetzt. Sogar einzelne Kommunen versuchten sich an verschiedenen Konzepten, um wirtschaftliches und gesellschaftliches Leben in Einklang mit der Virus-Bekämpfung zu bringen.

Angekommen im Superwahljahr 2021 bedeutet das politisch, dass die Umfragen ein sich immer weiter ausdifferenzierendes Parteiensystem andeuten. Mittlerweile liegt die Union in den Umfragen deutlich unter 30 %.^1^ Es fällt den merklich professioneller auftretenden Grünen unter der Ägide der beiden Parteivorsitzenden Annalena Baerbock und Robert Habeck zunehmend leichter, die Union nicht nur auf Länderebene herauszufordern oder zu schlagen – wie in Baden-Württemberg – sondern sogar auf Bundesebene.^2^ Neben den Grünen und der Union, die sich beide zwischen 23 % und knapp 30 % in den Umfragen bewegen, gibt es mit der SPD, den Linken, der FDP und der AfD vier Parteien, die sich zwischen knapp 8 % und knapp 17 % befinden. Klassische Volksparteien verlieren an Attraktivität und die plurale Gesellschaft wirkt mit zeitlicher Verzögerung nun auch auf die Parteienlandschaft. Konsequenzen können beispielsweise Vielparteienregierungen sein, welche häufig unter dem Verdacht stehen, instabiler zu sein.

Um einem solchen Superwahljahr mit den erheblichen politischen Implikationen und unter den besonderen Bedingungen gerecht zu werden, wurde diesmal ein besonders umfangreicher Schwerpunkt geplant. Beginnen wird Heike Merten mit einer juristischen Einschätzung von Wahlen in Pandemiezeiten. Anschließend widmet sich Salvatore Barbaro der Frage der Entbehrlichkeit von Stichwahlen bei Kommunalwahlen. Karl-Rudolf Korte erörtert sodann die Frage nach den Mobilisierungsmöglichkeiten in unsicheren Zeiten, wobei ein besonderer Blick auf die Möglichkeiten der etablierten Parteien gelegt wird. Dieser Analyse folgt ebenfalls eine politikwissenschaftliche Betrachtung. Arndt Leininger und Aiko Wagner diskutieren die Frage nach den Herausforderungen, die sich durch die Pandemie für die repräsentative Demokratie ergeben. Bekannt ist, dass die Corona-Pandemie in besonderer Art und Weise die Familien und die schulpflichtigen Kinder vor kaum zu meisternde Aufgaben stellte. Aus diesem Grunde plädieren Mathias Huebener und C. Katharina Spiess in ihrem Beitrag dafür, den Familien die Systemrelevanz zuzusprechen. Im folgenden Essay stellen Vera Freundl, Philipp Lergetporer und Larissa Zierow die desaströse Bilanz der deutschen Bildungspolitik während des vergangenen Jahres zur Diskussion. Ein Schwerpunkt zum Superwahljahr wäre ohne eine kommunikations- und medienwissenschaftliche Perspektive unvollständig. Diese wird eröffnet durch den Beitrag von Sebastian Jarzebski. In seinem Beitrag betrachtet er die Möglichkeit der Wahlkampfkommunikation unter Corona-Bedingungen. Matthias Degen betrachtet im letzten Beitrag des vorliegenden Schwerpunktes, wie der Journalismus mit der Krise umgegangen ist und inwiefern sie sich auf die Berichterstattung im Superwahljahr auswirken kann.

## Footnotes

1 Hier spielt sicher auch die Maskenaffäre eine Rolle, welche im Frühjahr 2021 die Union stark erschütterte. Auch der späte und von großer Zerstrittenheit zwischen den Schwesterparteien zeugende Kampf um die Kanzlerkandidatur zwischen Armin Laschet und Markus Söder führte zu einer weiteren Erosion von CDU und CSU.

2 Erste Umfragen deuten darauf hin, dass die Grünen deutlich zur Union aufgeschlossen haben. Manche Umfragen weisen die Grünen sogar vor der Union aus (siehe hierzu https://www.wahlrecht.de/umfragen/; Einsicht: 28.04.2021).

